# Preface to the fourth issue of Journal of Cardiovascular Disease Research

**DOI:** 10.4103/0975-3583.74257

**Published:** 2010

**Authors:** Jing Dai

**Affiliations:** *Academic Executive Editor, Journal of Cardiovascular Disease Research, Harvard Medical School, Boston, MA, USA. E-mail: jing.dai@jcdronline.org*

Cardiovascular disease is a leading cause of morbidity and mortality worldwide. The *Journal of Cardiovascular Disease Research* (JCDR) is honored to publish the fourth issue dedicated to cardiovascular disease research. The aim of this issue is to combine the various perspectives of clinical cardiology as well as basic research in this important discipline of medicine. Original papers, case report-based clinical studies and invited review articles are presented to the readers of JCDR.

In this issue, Dr. Caldwell from the Department of Pharmacology and Toxicology, Medical College of Georgia, has reviewed the role of RhoA/Rho kinase (ROCK) pathway in endothelial dysfunction. Endothelial dysfunction is a key event in the development of vascular disease and precedes clinically obvious vascular pathology. Abnormal activation of the RhoA/ROCK pathway has been found to elevate vascular tone through unbalancing the production of vasodilating and vasoconstricting substances. Inhibition of the RhoA/ROCK pathway can prevent endothelial dysfunction in a variety of pathological conditions. This review, based on recent molecular, cellular and animal studies, focuses on the current understanding of the ROCK pathway and its roles in endothelial dysfunction. In the second review article, Dr. Saleem *et al*. have summarized that moderate consumption of red wine prevents cardiovascular disease through several mechanisms, including increases in the high-density lipoprotein-cholesterol plasma levels, decreased platelet aggregation, antioxidant effects and restoration of endothelial function. They discuss that red wine possesses a diverse range of biological actions and may be beneficial in the prevention of cardiovascular disease.

Highlights in this issue are case report-based clinical studies. The majority of clinical practice relies on the accurate synthesis and extrapolation of the patient’s history, physical examination and laboratory evaluation. Chetwood *et al*. reported a very unusual case of a 35-year-old obese male patient with a left ventricular thrombus secondary to a silent myocardial infarction and resultant shower emboli to multiple arterial sites. This rare and extreme example of a left ventricular thrombus in a young male patient emphasizes the potential sequelae of the condition. Saritas *et al*. presented several cases about the management of aortico-left ventricular tunnel. Wang *et al*. studied the modification of atrioventricular node in a special condition treating paroxysmal supraventricular tachycardia. They found that, in some special cases, modification of the atrioventricular node should rely on not only the junctional rhythm to determine the ablation effect but also on the cardiac electrophysiological examination.

In the original articles of this issue, Nasr *et al*. showed allopurinol and global left myocardial function in heart failure patients. Allopurinol did not produce significant clinical and functional improvements in unselected patients with moderate-to-severe heart failure. However, it suggested that it was useful in patients with elevated serum uric acid (SUA) in a manner according to degree of SUA reduction. SUA may serve as a valuable biomarker to target heart failure therapy. Morgado *et al*. conducted a cross-sectional survey in a hospital hypertension outpatient clinic located in the Eastern Central Region of Portugal. A total of 197 patients meeting the inclusion criteria and consenting to participate completed the interview. They found that poor medication adherence, lack of information about hypertension and side-effects should be considered as possible underlying causes of uncontrolled blood pressure (BP) and must be addressed in any intervention aimed to improve BP control. Saritas *et al*. evaluated the methods of percutaneous transcatheter interventions for combined congenital heart disease and its efficacy in children. They concluded that multiple transcatheter interventions in the same session were feasible, safe and effective, with satisfactory good results. A second intervention may be performed as a complementary procedure or independently to the first intervention.

JCDR is now indexed with PubMed. We would like to thank all the reviewers for their excellent work and the authors for their contribution. We expect that JCDR will soon be indexed with SCI, which will provide a higher platform for the authors and the readers, with a comprehensive overview of the most recent developments in cardiovascular disease research.

